# Female Goldeneye Ducks (*Bucephala clangula*) Do Not Discriminate among Male Precopulatory Display Patterns

**DOI:** 10.1371/journal.pone.0057589

**Published:** 2013-03-14

**Authors:** Benjamin Dane, Rebecca Harris, J. Michael Reed

**Affiliations:** Department of Biology, Tufts University, Medford, Massachusetts, United States of America; CNRS, Université de Bourgogne, France

## Abstract

Female goldeneyes remain motionless on the surface of the water while single males circle them performing a series of highly stereotyped displays. After performing between eight and 90 of these displays the male either copulates or attempts to copulate with the female. However, females allow only 58% of males to mount them, while rejecting 42%. We have examined 804 of these precopulatory sequences containing 11,841 actions in an effort to determine why females find some display sequences of males unsuitable, while others are accepted. Males have an extraordinarily varied sequence of actions, and sequence variation leading to successful and unsuccessful copulation attempts was similar. Most surprising was the tendency of males to eliminate one of the five actions, whether in successful or unsuccessful attempts. As unlikely as we think it might be as the result of natural selection, the only statistically significant difference we found between successful and unsuccessful attempts was the reduction in the frequency of expression of one or more of the behaviors in successful attempts. These observations, coupled with the large variation seen in most sequences, suggest that there is not a correct sequence, or even a correct set of actions leading to copulation. The male must, however, perform goldeneye species-specific precopulatory behavior as performed by adult males, although it apparently can be performed in a wide variety of patterns.

## Introduction

The courtship displays of ducks have long attracted the attention of ethologists both as a means of further elucidating evolutionary relationships and to understand the function of stereotyped signals ([1] in [2],[3]–[4]). Not only have signals proven to be highly stereotyped, but also to form a remarkably complex communication system. Such signals are conventionally divided into those seen in groups of birds, those that precede copulation, and those that follow copulation [5]. While those that are seen in groups are of bewildering complexity, sometimes involving dozens of individuals, those that precede and follow copulation involve only two individuals and therefore lend themselves more readily to analysis. Here we examine the specific sequence of behaviors preceding copulation and copulation attempts in goldeneye ducks. We found, much to our surprise, that females are remarkably indiscriminate in their choice of acceptable male pre-copulatory display patterns.

Other aspects of copulatory behavior in ducks, specifically anatidae, have been studied, such as: forced copulation [6], repertoire size [7], multiple functions of displays [8]–[10], and post-copulatory displays [11]. Payne and Pagel [12] discuss the selective advantage of repeating displays, while Rowe [13] reviews the advantages of signals having multiple components.

While the classic papers of Lorenz and the work of individuals such as McKinney have focused attention on the broader question of the ultimate function of displays, attention has recently been given to the proximate causation of displays, particularly to the question of the signal function of each display element. The function of such signals has been well reviewed by Searcy and Nowicki [14], while specific proposals for their function have been made by Grafen [15], Kirkpatrick and Ryan [16] and Ryan [17]. There is general agreement that the signals serve quite different purposes for the two sexes, particularly where pair bonds are not permanent. While males endeavor to mate with almost any female, females must endeavor to determine which males are of the highest quality. Assuming that producing the signals is costly, both in terms of energy and conspicuousness, males signal only enough to attract females. So “the interests of the signaler and receiver diverge” [14]. Grafen [15], with the aid of a mathematical model, proposed that a signaling system is evolutionarily stable when a male's signals are well correlated with his quality. Thus, he proposes, males of high quality signal more than do those of low quality. Consequently there should be strong selection for female discrimination and stereotyped male pre-copulatory displays should be useful signals to females. That such is the case has been shown in both a wide variety of studies and in a wide variety of taxa, particularly oscine song birds (reviewed by [14]). For example, village indigobird (*Vidua chalybeate*) females favor males with a high rate of song [18], female song sparrows (*Melospiza melodia*) favor males with more complex songs [19]–[21], female sedge warblers (*Acrocephalus schoenobaenus*) with males with a large repertoire [22]–[23] and female American goldfinches (*Carduelis tristis*) males are a bright yellow color [24].

Females may receive both direct and indirect benefits from chosen males. Direct benefits would include increased fecundity, while indirect benefits would include “good genes” for her offspring. Experimental evidence for these benefits has been elegantly investigated by Marion Petrie with a population of captive, but free-ranging, peacocks in an English park. She studied the birds over many generations and deduced that the most “elegant” males were both most successful at attracting females and in providing genetic material most likely to ensure the survival of offspring [25]–[26].

We have investigated female choice in goldeneye ducks. Females of this species respond to the pre-copulatory display of males in three ways: they may accept the male and allow him to mount, they may reject the male by diving away from him (or attacking him), or they may apparently “lose interest” in the male's display and swim away from him [27]. Males who are rejected may continue to display and once again attempt to copulate, and they may do so many times. Females who choose to reject males after the first bout of display may accept this attempt after subsequent display sessions. (Males frequently attempt to mount females without performing the normal series of pre-copulatory displays, but they are invariably rejected by the female diving or attacking the male [27].

Courtship displays of eiders (*Somateria* spp.) were analyzed in great detail by McKinney [28]. His exhaustive study of frequency and ordering of movements shows that in two races of eiders there are some changes in the frequency of actions over time and a slight tendency for ordering to take place. Similar conclusions for goldeneyes were reached for group behavior by Lind [29], Dane & van der Kloot [27], and Afton & Sayler [30]. Dane & van der Kloot [27] also studied pre-copulatory and copulatory behavior in detail and found evidence of stereotypy, frequency differences in display elements (but only slight linkage between elements), and possible changes in frequency as pre-copulatory and copulatory behavior approached copulation. Surprisingly, however, these earlier analyses report no evidence of the particular aspects of pre-copulatory behavior that were more likely to be found acceptable by females. Instead, almost any combination of displays, performed in almost any pattern, was accepted by females. In as much as male displays are costly in terms of time, energy, and conspicuousness, natural selection must benefit males who perform in a manner most likely to lead to female acceptance. Since all of the data in the first analysis were lost (see methods), one of us (B.D.) collected a new, much larger set of data in the hope of revealing the basis on which females make their choice.

In this new analysis, we have focused our attention on three questions: first, is each pre-copulatory display element critical to successful copulation; second, can analysis of successful compared to an unsuccessful pre-copulatory behavior reveal the function of each element; and third, do particular patterns of behavior play a role in female choice. Although this analysis was confined to study of behavior on the wintering grounds (the non-breeding season), the behavior could well form the basis of more permanent pair bonds on the breeding grounds.

## Methods

### Background on common goldeneye courtship

In common goldeneyes, courtship consists of two largely separate types of behavior: flock display (or social courtship) and precopulatory display [31]. In the first, large numbers of birds (3–40 individuals) perform a total of 18 different actions; 14 by the male and 4 by the female. Each bird may perform an action every few seconds leading to a highly complex and seemingly disordered displaying group. This activity in itself never leads to an attempt by a male to copulate with a female. However, one of the activities of flock display does directly lead to the second type of behavior (precopulatory behavior). This is when a male performs the ticking display (turning his head from side to side as he swims rapidly), usually alternated with head-throws, and leads a female out of the displaying group. If he succeeds, he radically changes his behavior, by performing the five main precopulatory behaviors: the display drink, the bill shake, the head rub, the head flick, and the wing stretch. All of these closely resemble comfort movements. No sounds are produced as the male performs these movements. (All of these displays are described in detail in [27], [31]). While the male is displaying, the female assumes the prone posture: she lies flat on the surface of the water and only very rarely makes any discernable movement. After the male performs between 8 and 90 of these displays (drink, bill-shake, head-flick, head-rub, wing-stretch), the male usually initiates a copulation attempt. Here he performs a unique set of actions not observed at any other time, and performs each of these only once: the crescendo (consisting of rapidly repeated bill shakes), display preen, and precopulatory steaming. Following a successful copulation, the male performs another novel set of actions (usually three, occasionally two) while the female performs one.

### Field Methods

Between 1956 and 1975, an unknown number of sequences (approximately 600–700) of precopulatory behavior were recorded on either movie film or with a tape recorder. A devastating fire at Tufts University in 1975 destroyed all of these data and their records with the exception of 5 sequences. Starting in 1975 and ending in 1985 799 new sequences were recorded (all by B.D.). The 804 total sequences contained 11,841 of the main precopulatory actions. Thirty-one sequences were recorded on 16 mm film using a 600 mm lens, and 773 sequences on a tape recorder while viewing the birds through a 30× spotting scope. Both the sequence of displays, and the interval between displays, were recorded. The accuracy of the tape recordings was tested by tape recording from movie film and checking these against frame-by-frame analyses of the movie film to evaluate the consistency of the results. No errors (in transcribing the filmed displays) were found in these tests, so it is assumed that the tape recordings were accurate.

All of the data were recorded in the Merrimack River basin at Newburyport, Massachusetts, USA. Most were recorded between January and April, although a few sequences were recorded in December. Many hundreds of goldeneyes winter on the Merrimack and there were usually several hundred under observation. No birds were marked, but considering the number under observation, it seems unlikely that a few individuals dominated the data set. (When water conditions were calm, as many as 20 pairs were seen to be simultaneously performing precopulatory behaviors.) This assertion is further reinforced because the birds drift up or down the river with the tidal surge and as a consequence are constantly flying up or down the river to get to the observation area, where they feed and court.

Out of the 804 total sequences, 289 were long enough (8 actions or longer) for the primary analysis. None of these sequences was broken up by interferences from other birds. The 289 “primary” sequences were divided into three different categories according to the outcome of the sequence. The most common outcome was when the pair successfully copulated and performed postcopulatory displays (referred to here as successful copulation). The second most common outcome was when the pair failed to even attempt to copulate, but instead started to engage in some other activity, usually starting to feed, preen, or to swim or fly away from each other. In these cases there was no discernable reason for the cessation of precopulatory behavior; the pair simply “breaks up.” A less likely outcome was when the male apparently performed normal precopulatory behavior, including the three specific actions that always precede successful copulation, but was unsuccessful in his attempt to mount the female (“unsuccessful copulation”). We paid particular attention to differences found between pairs that successfully copulated and pairs that attempted, but failed, to copulate.

These 289 “primary” sequences were subdivided into two subsets; one subset of 173 shorter sequences containing 8–19 actions each, and one subset of 116 longer sequences containing 20 or more actions each. The selection of a cutoff between 19 and 20 displays was somewhat arbitrary, but designed to balance sequence length with sample size (number of sequences). Of these 116 “long” sequences, 67 led to copulation, 25 to unsuccessful copulation, and 24 to breaks up. The other 515 sequences (of the 804 total) were of short duration due to interference from other birds (usually males; referred to as “broken up”), because the birds stopped displaying (“breaks up”), or because precopulatory behavior was detected (and recorded) close to the end of the sequence. These short sequences were particularly helpful in analyzing the start and end of precopulatory behavior.

In conducting our analyses we used the sequences in the following manner, but in all cases relied most heavily on the 289 primary sequences. Examining the frequency of display actions in each category and the changes in frequency as the sequence progressed we used the 289 primary sequences. Analyzing the start of precopulatory behavior, unsuccessful copulation, copulation attempts that subsequently succeed, and the timing of actions we used any of the 804 sequences that were applicable.

Previous work had established that the five main precopulatory actions occur with different frequencies and that these actions have a slight tendency to be linked in pairs [27] so these aspects of display were not analyzed again. We asked, instead, four questions in the analysis, all with the aim of trying to elucidate what aspects of male and female behavior determine the pairs' chances of successfully copulating.

1. Is the frequency of each action the same in each precopulatory category (successful, unsuccessful, and “breaks up”)?
2. Is there a specific start or end (an action or a series of actions) in each sequence? (This question refers only to the five main precopulatory actions, not to the invariable series of three unique actions that precede any copulation attempt.)
3. Does the frequency of each action change as the sequence progresses from start to finish? More particularly, does the male alter his behavior after he attempts, but fails, to copulate, and subsequently succeeds? These behavioral sequences are particularly curious, since it is possible that the male alters his strategy if his initial attempt(s) is (are) rejected by the female
4. Does the frequency at which actions are given correlate with precopulatory success or failure?

## Results

### Frequency of display actions in each category

Table 1 lists the frequencies for each of the main precopulatory actions in each category for common goldeneye, as well as their overall frequency. The data are taken from the 289 sequences (those that contain at least 8 actions each). Drinking and head-flicks made up over half of the behaviors in both successful and failed copulation attempts.

**Table 1:** Frequency of display actions in each category, from 289 behavioral sequences.

Action	Successful copulation	Unsuccessful copulation	Break-up	Total
Drink	788 (27%)	219 (31%)	268 (29%)	1275 (29%)
Head-flick	769 (26%)	210 (30%)	177 (19%)	1156 (25%)
Bill-shake	517 (18%)	77 (11%)	191 (21%)	785 (18%)
Wing-stretch	513 (18%)	107 (15%)	145 (16%)	765 (17%)
Head-rub	262 (9%)	80 (12%)	128 (14%)	470 (11%)
Total	2849	693	909	4451

### The start of precopulatory behavior

Of the 804 sequences where the start of precopulatory behavior was observed, there were 51 instances when males who were engaged in flock display led females out of the group and started precopulatory display. (A spreadsheet that includes all of the sequences can be downloaded at http://ase.tufts.edu/biology/labs/reed/publications/supplementary.htm.) So for all of these sequences we know which actions initiated precopulatory display. In 21 of these instances, the pair copulated (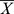 = 25 actions – range 10 to 47, S.D. = 11.1), in 9 they attempted but failed to copulate (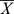 = 29 actions – range 12 to 78, S.D. = 19.6), in 11 the pair breaks up, and in 10 they were broken up by interference from other birds. Table 2 shows the frequency of starting actions as well as the frequency of occurrence of each in the subsequent 5 actions in each sequence. Again, drinking was the most common behavior, and it was significantly more common than expected from random (X^2^ = 133.1, df = 4, p<0.001). In addition, the first action in a sequence disproportionately favored drinking over other actions compared to the subsequent five actions (X^2^ = 52.6, df = 4, p<0.001) (Table 2).

**Table 2:** Actions that start precopulatory behavior regardless of outcome.

Action	First Action	Next Five Actions
Drink	42 (82%)	68 (30%)
Head-flick	4 (8%)	61 (27%)
Bill-shake	1 (2%)	27 (12%)
Wing-stretch	0	35 (15%)
Head-rub	4 (8%)	35 (15%)

Data come from the 51 sequences where the start of pre-copulatory behavior was observed. First Action is the frequency with which the action initiates the sequence. Next Five Actions is the number of times the action occurs among actions two through six in the sequence.

Note: Since some sequences were broken up or discontinued after starting precopulatory behavior, actions two to six add up to only 226 actions, instead of 255 actions.

### Changes in frequency as the sequence progresses

**Figure 1:**
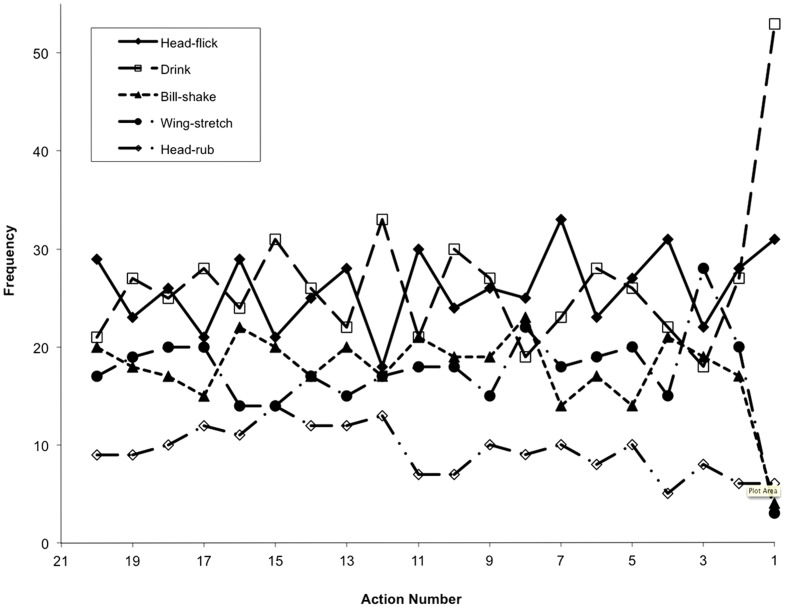
The frequency distributions of five specific actions in sequences leading to copulation from action 20 (that nearest the start of the sequence for long sequences and action 8 for the short sequences) to action 1 (that at the end of the sequence). The data are taken from the 189 primary sequences leading to copulation.

**Figure 2:**
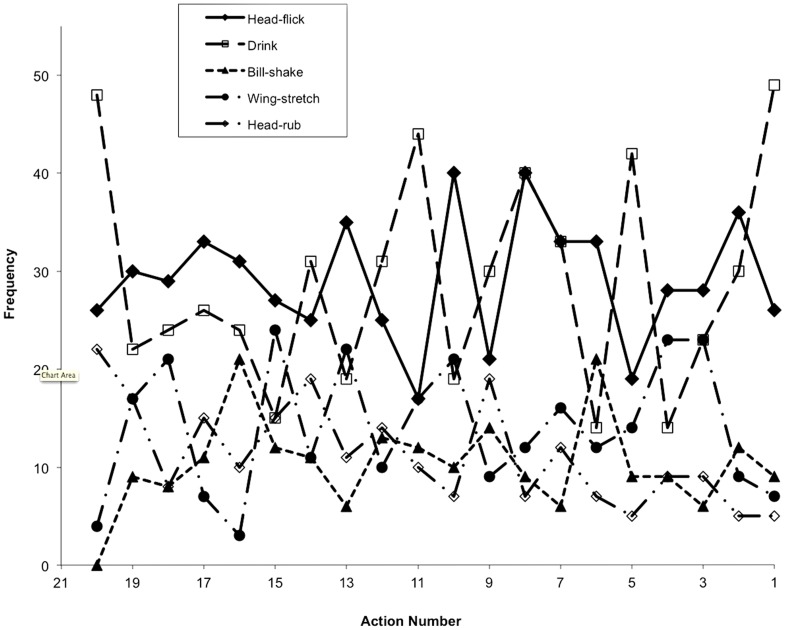
The frequency distributions of five specific actions in sequences leading to failed copulation attempts from action 20 (that nearest the start of the sequence for long sequences and action 8 for the short sequences) to action 1 (that at the end of the sequence). The data are taken from the 43 primary sequences leading to failed copulation attempts.

Figures 1 and 2 show the frequency distributions of the five specific actions in sequences leading to copulation or unsuccessful copulation from action 20 (that nearest the start of the sequence for long sequences and action 8 for the short sequences) to action 1 (that at the end of the sequence). The data are taken from the 289 primary sequences.

In successful copulation sequences (189 total) the frequency of all 5 actions remains fairly constant until the last action – here the proportion of behaviors that were drinks increases significantly from an average of 27% to 53% (exact binomial text, p<0.001). In unsuccessful copulation sequences (43 total) there is great variation in frequency for both drinks and head-flicks, while bill-shakes, head-rubs and wing-stretches remain relatively constant. Once again, however, the frequency of drinks rises significantly for the final action to 49% of the actions (exact binomial text, p<0.001). We also note that there is a negative relationship between the frequency of head flicks and drinks, the two most common actions, particularly for series from successful copulations (Figure 1). In breaks-up sequences (57 total) head-flicks, bill-shakes, head-rubs, and wing-stretches remain constant, but the frequency of drinks show two peaks centering on actions 11 and 16 (data not shown). However, it must be remembered that the end of these sequences is unpredictable; the birds simply stop displaying, so that the timing of sequences and the position of each action is arbitrary.

We looked for a difference in sequences among outcomes using logistic regression for successful outcomes (n = 189) vs. failed attempts (n = 43) and failed attempts vs. breaks up (n = 54; sequences leading to breaking up without a copulation attempt), using the logistic procedure in SAS (v. 9.1.3). For both models we did two analyses, one where our independent variables included the proportions of each of the actions within each sequence, and one using the absolute frequencies of the behaviors. In the first analysis we separately included the crescendo just prior to the copulation attempt, but in the latter analysis it was excluded because behavioral sequences that ended in a break-up before copulation did not have crescendos. For all analyses, data were evaluated for violation of model assumptions and they conformed to test requirements. Comparing successful with unsuccessful copulation attempts, copulation success was weakly associated with lower proportions of drinks and head rubs (for both, 0.05<p<0.10; maximum re-scaled r^2^ = 0.14 for the entire model). Results were similar for the frequency of behaviors, with more bill shakes and fewer drinks and head rubs associated with successful copulations, but with low explanatory power (for each, p<0.01; maximum re-scaled r^2^ = 0.18 for the entire model). The other independent variables were not significantly different between the treatment groups in either analysis. The differences between failed copulation attempts, and attempts that ended in a break-up before a copulation attempt, were slightly stronger for frequency of behaviors, where there were more head flicks associated with failed attempts (p<0.01; maximum re-scaled r^2^ = 0.28 for the entire model); a weaker result was found when proportions were analyzed, with none of the proportions showing significant differences between treatments (p>0.1 for each; maximum re-scaled r^2^ = 0.20 for the entire model)

We also looked to determine if there were patterns within the sequences of precopulatory behaviors. This was done using multinomial outcome analysis, which can be thought of as logistic regression with multiple outcomes where data are serial. We treated a bird's sequence data, i.e., a time series of behaviors, as the observation, and looked at the transition probabilities between behaviors to look for patterns in the sequences. In the multinomial outcome analysis of sequence data, bird display behaviors were modeled as a sequential discrete outcome multinomial process with 5 possible outcomes ( = the displays). A given sequence was considered correlated and corresponding multinomial probabilities were allowed to have within category and across category probability dependence structure (autoregressive and cross lag effects). Bird sequences were pooled within treatment category for model estimation, and it is assumed that pooled sequences within treatment are exchangeable, so long as within bird sequence order is preserved. Each of the cell multinomial probabilities were modeled on the log scale as a linear combination of lag(1) autoregressive effect and cross category effects including the treatment effects. Bayesian estimation was performed with WinBugs software using non-informative Gaussian priors on all parameters via Monte Carlo Markov Chain sampling. A burn in sample of length 20,000 was used with a follow up of 30,000 for chain convergence and estimation. The last 10,000 observations were thinned every 5 to reduce parameter autocorrelation, resulting in 2,000 observations for posterior distribution summary purposes. (Similar results occurred with longer summary observations.) The analysis showed no statistically significant differences between treatments (successful copulation, failed copulation attempt, breaks up); that is, all of the confidence regions included zero.

Finally, to further investigate whether the male might be altering his behavior during precopulatory display, we analyzed the long copulation sequences using several approaches. First, to generally describe the data, we divided the long copulation sequences in half, starting each individual sequence at the last action before the copulation attempt, and looked at the frequency of actions performed in the first and second half of all long sequences. (The data set for unsuccessful copulation sequences is too small to allow such a comparison.) A total of 2010 actions were performed in the 67 of 116 long sequences that led to successful copulation. Although the relative frequencies of the actions differ significantly between the first and second half of the series (X^2^ = 18.2, df = 4, p = 0.001), the differences appear to be minimal, with slightly more bill-shakes and head-rubs and fewer wing-stretches in the first half (Table 3). Note that crescendos can occur in the body of the display sequence, and not just at the end. This is true because males sometimes perform this action interspersed with the five main displays.

**Table 3:** Frequency of actions in the first and second half of long successful copulation sequences (n = 67 sequences).

Action	Actions in first half	Actions in second half
Drink	275 (27%)	280 (28%)
Head-flick	230 (23%)	271 (27%)
Bill-shake	227 (23%)	188 (19%)
Wing-stretch	125 (12%)	148 (15%)
Head-rub	147 (15%)	99 (10%)
Crescendo	4	16
Totals	1008	1002

### Unsuccessful Copulation

Unsuccessful copulation attempts were analyzed in an effort to determine why the pair had been unsuccessful. A total of 148 attempts by 93 individuals were recorded, including attempts by males who were ultimately successful. Of the individuals that never succeeded, 65 attempted and failed copulation once, seventeen made two attempts, one made seven attempts, and one made nine attempts. The majority of these failed attempts were due to female rejection (Table 4). Females rejected males most often by diving when the male attempted to mount (n = 109; 74%) or by rising out of the prone position before the male reached them (n = 5; 3%). In other instances, females swam away from the male as he “steamed” toward them, or they attacked him (n = 8; 5%). Males also caused failures. Most often they failed to grab the back of the female's head, and thus could not maintain their position (n = 11; 7%). In a few cases, males simply failed to reach the female (n = 2; 1%). In 13 instances (9%) the reason for failure was not apparent.

**Table 4:** Frequency of last action before unsuccessful copulation attempts.

	Female rejection	Male failure	Unknown cause
Number of observations	71	13	13
Frequency of last action			
Drink	55%	63%	69%
Head-flick	29%	22%	31%
Other actions	16%	15%	0%

These data were derived from the 93 of 804 sequences that were applicable.

To understand the reasons for failure we looked at the last action before an attempt. We found no relationship to female rejection or male failure (X^2^ = 2.62, df = 4, p = 0.6) (Table 4). To avoid biasing the data, each male making multiple attempts is given an average value. Since multiple attempts can include both female rejection and male failure, the total number of cases is 97 not 93.

These data can also be analyzed to compare the behavior of the male as he makes consecutive, unsuccessful attempts, but is never successful. There were 23 instances where males made two or more unsuccessful attempts (17 that never copulated and 4 that were eventually successful). Seventeen made two attempts, one made three, one made four, three made five and one made seven. Again, the small data set only allows the analysis of the final two attempts. Data for the final action performed and the number of actions performed in each sequence showed no apparent differences between the penultimate and ultimate attempts, although sample sizes for these tests were small so statistical power was low (Table 5).

**Table 5:** Frequency of the last action in two consecutive unsuccessful copulation attempts (n = 17 sequences) and between the final unsuccessful copulation and subsequent copulation (n = 4 sequences).

Action	Penultimate attempt	Ultimate attempt	Final unsuccessful attempt	Copulation
Drink	10	10	4	7
Head-flick	6	8	6	3
Bill-shake	0	0	0	0
Wing-stretch	2	0	1	1
Head-rub	2	0	0	0
No. of actions before crescendo	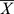 = 12.6 (SD = 10.66)	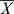 = 9.0 (SD = 9.67)	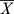 = 7.5 (SD = 4.0)	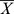 = 11.9 (SD = 12.7)

The penultimate and ultimate attempts refer to sequences where birds do not successfully copulate; the final unsuccessful attempt and copulation columns refer to birds that eventually copulate successfully (n = 11 sequences).

### Copulation attempts that subsequently succeed

There were 11 instances when pairs that attempted, but failed, to copulate, subsequently succeeded. In six instances, the male made one attempt before succeeding, in two instances he made three attempts before succeeding, and one instance each of four, five, and nine attempts before succeeding. Once again, an analysis of the last action and the number of actions was undertaken. The small data set only allows a comparison between the final (or only) attempt and that before copulation. The data show no obvious differences (Table 5).

### Differences in display frequency and form

Since the birds were not marked, there is no information on the consistency of each individual's behavior relative to its success in securing copulations. It was possible, however, to analyze the different patterns of behavior seen in both successful and unsuccessful pairs. This analysis was again confined to the long sequences. In a surprisingly high percentage of the 67 successful copulatory sequences, males eliminated one of the five main precopulatory displays, without apparent effect on their rate of success. Of these sequences, there were 24 (36%) where the male left out one of the actions completely and in 57 (85%) one of the actions were given less than 5% of the time (Table 6). Of the 25 unsuccessful copulation sequences there was 1 (4%) where the male left out one action completely, and 6 (24%) where one of the actions was given less than 5% of the time. This pattern was significantly different (X^2^ = 15.2, df = 1, p<0.001). Data for sequences that lack an action, or exhibit low frequencies of actions are shown in Table 6; sample sizes for statistical tests using these data were small so statistical power was low. There were also sequences in both unsuccessful and successful copulation where males performed actions with unusually high frequency. There were 19 successful copulation sequences (out of 67) and five (of 25) unsuccessful copulation sequences where one action occurred at more than twice its expected frequency based on random expectations, and these are not significantly different (X^2^ = 0.3, df = 1, p>0.50) (Table 6).

**Table 6:** Frequency of 116 long copulation and unsuccessful copulation sequences lacking one action type, with one action type given <5% of the time, or with action types of notably high frequency.

	Successful copulation			Unsuccessful copulation	
Action	Lacking action	Low frequency	High frequency	Lacking or low frequency	High frequency
Drink	1	0	0	0	0
Head-flick	4	5	2	0	0
Bill-shake	4	12	11	3	1
Wing-stretch	0	6	0	2	1
Head-rub	15	10	6	2	3
Total	24	33	19	7	5

The form of actions also differs, but rarely. The aberrances most commonly seen were when two actions were combined, such as head-rubs and drinks, and bill-shakes and drinks. Out of the 804 precopulatory sequences there were 7 sequences with abnormal actions; 3 were in successful copulation sequences, 3 in breaks-up-on-own sequences, and 1 in an unsuccessful copulation sequence.

## Discussion

Having examined the 804 sequences of precopulatory behavior from various perspectives, we are still far from ascertaining what aspects of the male's behavior determines whether or not a copulation attempt will be successful. The following aspects of male behavior are similar whether he succeeds or fails: (1) the start of precopulatory behavior almost always begins with a drink; (2) the last action before the crescendo is drink in at least 50% of all sequences; (3) the form and duration of drink actions is the same, whether at the start or at the end of the sequence; and (4) the rate at which he performs the actions is the same. In contrast, other aspects of the male's behavior differ between successful and unsuccessful copulation attempts. (1) The overall frequency of actions differs significantly in the three categories of outcome. (2) In copulation sequences the frequency of each action type stays the same during the whole sequence, whereas in unsuccessful copulation the frequency of drinks and head-flicks is highly variable. McKinney [28] reported similar changes in frequency of actions in the precopulatory display of eiders. (3) Finally, whether males are successful or unsuccessful, there is large variation in their individual behavior. Actions can be left out entirely or performed at unexpectedly low or high rates whether he succeeds or fails. Such aberrances are more common in successful sequences than in sequences when he fails.

Of particular interest are instances when the male repeatedly attempts, but fails, to copulate, and sequences when he attempts (often repeatedly) to copulate and finally succeeds. In the case of repeated failures we wondered if the male would change his behavior. Such an assumption seems particularly reasonable because failures are usually caused by the female diving when the male attempts to mount her. Yet, the male does not change his behavior. The number of actions he performs before any attempt remain the same, and he performs a drink as the last action in the sequence with the same frequency (see also [27]).

What of sequences where the male repeatedly fails and then finally succeeds in his copulation attempts? Once again, his behavior remains basically the same. There is one sequence when the male made four unsuccessful attempts before succeeding on the fifth attempt. This male performed precisely the same sequence of actions each time (even the interval between actions was similar), but on the fifth attempt he was successful.

There were some slight differences between the behavior of males who succeed and those who fail. Specifically, there were more omitted behaviors and lower variance in frequencies of the various per-copulatory actions in successful copulations than in failed attempts. We wonder, though, about the biological, as opposed to statistical, significance of these differences. The differences observed do not have apparent biological significance, unless the latter somehow correlates with male quality or motivation. Considering the enormous variability in the behavior of males who succeed, or those that fail, is it reasonable to assume that success or failure depends on small differences in the frequency of performance of some actions? If males often succeed while behaving in ways that differ markedly from average behavior, should other behavioral differences be those that cause the female to reject him? Perhaps most surprising of all is the fact that most “long sequence” males who are successful leave out one action entirely or perform the action at very low frequency. Yet such seemingly aberrant behavior (possibly individual differences) appears to confer no disadvantage. McKinney [5] proposed that “It seems unlikely that each display could be transmitting a specific message to the female;…” (p. 241), and that instead he was transmitting information about his overall motivation.

The pair bond formed on the wintering grounds may be brief, but it still represents the female's choice of a mate; however temporary it may be. Her choice clearly reflects, in part, the physical appearance of the male since sub-adult males are invariably rejected [27]. Sub-adult males lack the striking black and white body plumage and the iridescent green head plumage of adults, yet they perform the same basic precopulatory suite of actions performed by adults. These individuals regularly direct their precopulatory behavior at females, even going so far as to “steam” toward females. Yet in 55 years of observation no female was ever seen to allow an immature male to mount her. Thus performing the proper set of precopulatory actions is not enough to induce females to accept them; they must also have the proper species-specific appearance of adults.

While sexual selection favors females who show great selectivity in choosing a mating partner [32]–[33], females who reject males usually do so after being subjected to a long bout of display during which they are vulnerable to predators. Females could be basing their choices (in part) on factors such as dominance rank or experience [34], or possibly close genetic relatedness [35], which could not be evaluated in this study.

Finally, there is the question of the possible role pair formation might have on the outcome of precopulatory behavior. It is possible that successful pairs are those that have achieved pair formation, whereas unsuccessful ones are unpaired. Having watched common goldeneye behavior during two seasons on the breeding grounds (B.D. 1958 and 1962) this possibility seems unlikely. First, both males and females are constantly changing partners during courtship on the wintering grounds. Males often copulate with one female, and following copulation, join another courting group, or start precopulatory behavior with another female. Females seem equally fickle, constantly shifting from following one male to following another; the prelude to precopulatory behavior. Second, when the birds are truly paired, on the breeding grounds, their behavior is very different from that observed in this study. Courting groups do form, but they are dominated by aggressive behavior, often quite violent. The paired male and female attack other individuals, driving them away, and breaking up the courting group almost as soon as it is formed. They then often engage in precopulatory behavior and copulation. In so far as it is possible to keep track of individuals, they appear to remain with the other member of the pair, and successful copulation is the usual result of precopulatory behavior. Unsuccessful copulation almost always results from interference from other individuals, which ends in violent aggressive chases. Thus pairing, or lack thereof, seems an unlikely explanation for precopulatory success or failure on the wintering grounds.

We are left with the disquieting finding that the specific nature of the male's precopulatory behavior does not predict his chances of succeeding in any attempt to copulate. It seems instead that he can draw on a variety of strategies, any one of which may allow him to copulate. From our data, it appears that one set of behaviors may be successful, while another is not, but it seems that the successful set is highly variable and may be successful at one moment while failing at another. This may well be due to the state of the female's readiness, which clearly changes with time, and almost certainly reflects large individual differences.

Precopulatory behavior in common goldeneyes does not, then, seem to depend on a “correct” sequence of actions, or even on a “correct” set of actions. A male may perform many actions or few actions; perform all of the species-specific behaviors or only some of them; or perform them with the normal frequency or with a highly aberrant frequency. To be successful, however, he must perform goldeneye species-specific precopulatory behavior, but it can be performed in a wide variety of patterns.
